# Nuclear Imprisonment: Viral Strategies to Arrest Host mRNA Nuclear Export

**DOI:** 10.3390/v5071824

**Published:** 2013-07-18

**Authors:** Sharon K. Kuss, Miguel A. Mata, Liang Zhang, Beatriz M. A. Fontoura

**Affiliations:** Department of Cell Biology, University of Texas Southwestern Medical Center, Dallas, TX 75390, USA; E-Mails: Miguel.Mata@utsouthwestern.edu (M.A.M.); Liang6.Zhang@utsouthwestern.edu (L.Z.); Beatriz.Fontoura@utsouthwestern.edu (B.M.A.F)

**Keywords:** virus, influenza virus, vesicular stomatitis virus, VSV, NS1, matrix protein, nuclear export, nucleo-cytoplasmic trafficking, mRNA export, NXF1, TAP, CRM1, Rae1

## Abstract

Viruses possess many strategies to impair host cellular responses to infection. Nuclear export of host messenger RNAs (mRNA) that encode antiviral factors is critical for antiviral protein production and control of viral infections. Several viruses have evolved sophisticated strategies to inhibit nuclear export of host mRNAs, including targeting mRNA export factors and nucleoporins to compromise their roles in nucleo-cytoplasmic trafficking of cellular mRNA. Here, we present a review of research focused on suppression of host mRNA nuclear export by viruses, including influenza A virus and vesicular stomatitis virus, and the impact of this viral suppression on host antiviral responses.

## 1. Introduction

Nucleo-cytoplasmic trafficking of proteins and RNA is critical for proper cellular functions and survival. Genomic DNA is contained within the cell nucleus, which is compartmentalized from the cytoplasm by a nuclear envelope perforated with nuclear pore complexes (NPC) that regulate nuclear entry and exit of molecules. Cellular mRNAs are transcribed and processed within the nucleus prior to export to the cytoplasm for translation. For cells to respond to different stimuli and environmental cues, such as pathogen infections, transcriptional and post-transcriptional changes can occur within the nucleus to commence the appropriate cellular response. 

Regulation of cellular innate immune responses to pathogens occurs at multiple levels, including at the level of nucleo-cytoplasmic trafficking, whereby nuclear export of mRNAs encoding cellular defense proteins is critical for a host response to pathogen invasion. During a viral infection, particular pathogen associated molecular patterns (PAMPs) are recognized by cytoplasmic or endosomal pattern recognition receptors (PRRs). Host recognition of pathogens initiates autocrine and paracrine innate immune signaling to produce cytokines and chemokines that establish a pro-inflammatory, anti-proliferative antiviral state [1]. Type I (IFNα, IFNβ), II (IFNγ) and III (IFNλ) interferons (IFN) are antiviral cytokines critical for restricting viral infection by inducing expression of mRNAs that encode antiviral factors. DNA and RNA viruses alike are susceptible to IFN-induced cellular responses; therefore, they employ many strategies to block host up-regulation of antiviral response genes, including inhibition of both host transcription and post-transcriptional steps, which involves mRNA nuclear export and translation. 

All DNA viruses replicate within the nucleus except poxviruses, asfarviruses and phycoviruses. Conversely, very few RNA viruses, including bornaviruses, orthomyxoviruses and retroviruses, replicate in the nucleus [2]. Viruses that replicate in the nucleus must compete with host mRNAs to efficiently export their viral mRNAs from the cell nucleus to express proteins necessary for further replication and/or nascent virion assembly. This presents a dilemma for nuclear-replicating viruses as they require host nuclear export pathways to express their genes. 

**Figure 1:**
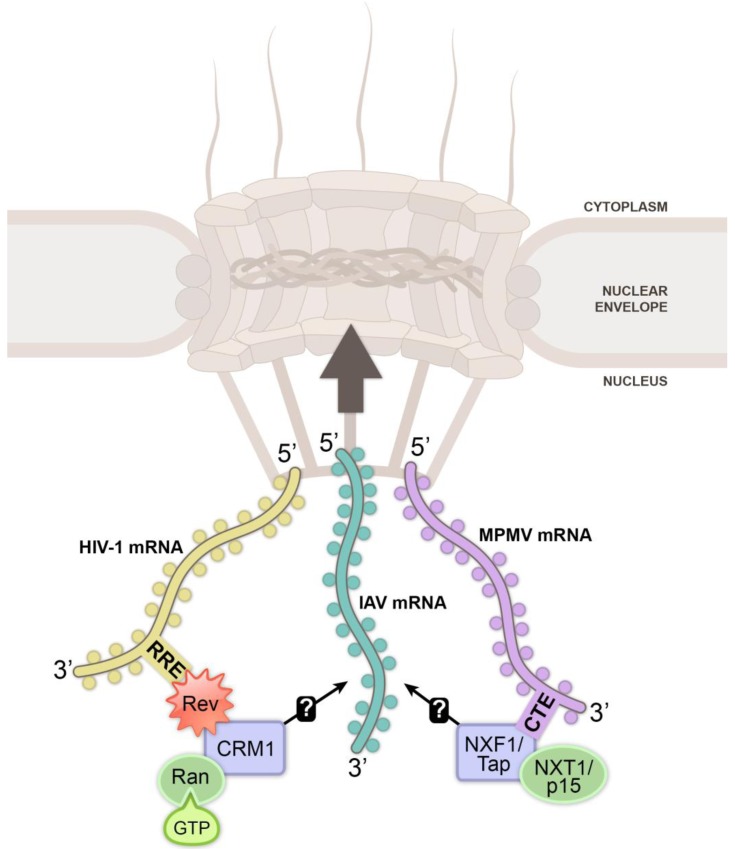
Viruses use cellular mRNA nuclear export pathways. The export receptor CRM1 was identified through its interaction with HIV-1 (Human immunodeficiency virus-1) Rev protein. Rev facilitates nuclear export of unspliced or partially spliced lentiviral mRNAs mediated by the Rev-responsive element (RRE), an RNA signature on lentivirus mRNAs. The RNA-Rev-CRM1 complex binds RanGTP, and the complex is then translocated through the nuclear pore complex (NPC). NXF1/Tap, which heterodimerizes with NXT1/p15, was established as a nuclear export receptor that is required for MPMV (Mason-Pfizer monkey virus) mRNA export. NXF1/Tap interacts with the constitutive transport element (CTE) on type D retrovirus mRNAs to promote their nuclear export. IAV (Influenza A virus) was reported to use both the CRM1 and NXF1/Tap pathways; however, it remains unclear exactly how IAV achieves nucleo-cytoplasmic transport of its own viral RNA. Nuclear export is depicted as the large arrow traversing the NPC, which is embedded in the nuclear envelope. Circles surrounding mRNAs depict RNA-binding proteins.

**Figure 2:**
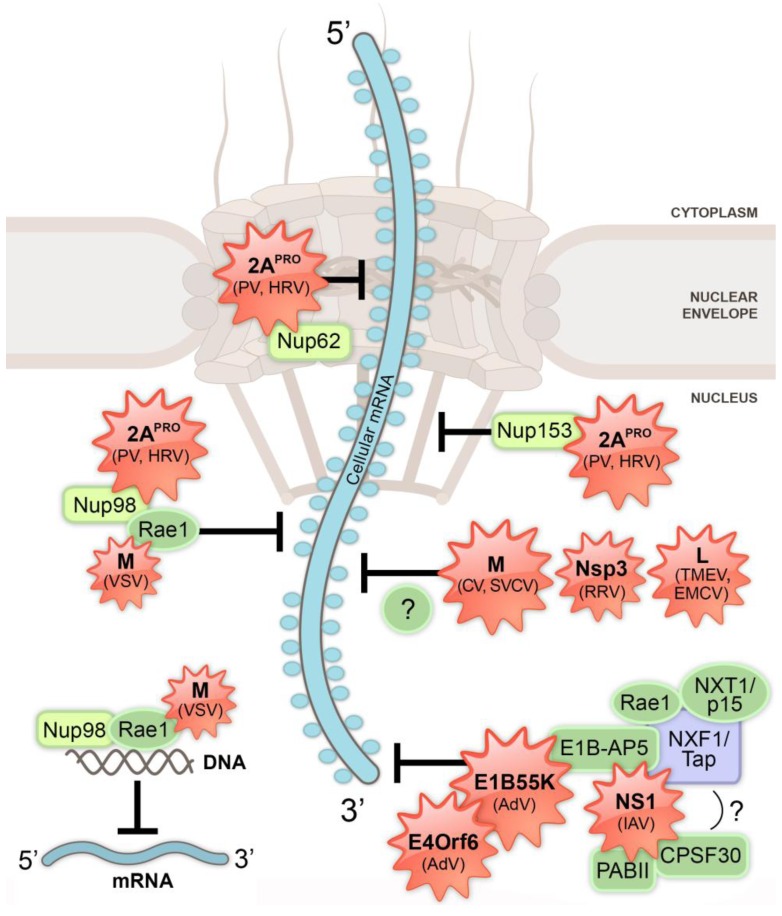
Multiple viruses target cellular mRNA processing and/or export pathways to prevent nuclear export of host mRNA. Processed cellular mRNA associated with RNA-binding proteins (circles encasing the mRNA) is exported from the nucleus through the nuclear pore complexes (NPC) embedded in the nuclear envelope. Viral proteins (red starbursts; viral protein name is bold and virus name is listed in parentheses) disrupt nuclear mRNA processing and export by interacting with host factors (green and purple). These effects promote host shut-off and perturb antiviral signaling, as described in the text. VSV M protein interacts with Rae1 and Nup98, resulting in mRNA nuclear export block. Additionally, this complex may be involved in transcription inhibition of a subset of genes. IAV NS1 binds and disrupts factors involved in cellular mRNA processing and export. CV, SVCV, RRV, TMEV and EMCV inhibit nuclear export of cellular poly(A) mRNA. AdV E1B 55K and E4Orf6 proteins inhibit cellular mRNA export likely by interacting with E1B-AP5 and NXF1/Tap. Abbreviations: PV, poliovirus; HRV, human rhinovirus; VSV, vesicular stomatitis virus; IAV, influenza A virus; AdV, adenovirus; CV, chandipura virus; SVCV, spring viremia carp virus; RRV, rhesus rotavirus; TMEV, Theiler’s murine encephalomyelitis virus; EMCV, encephalomyocarditis virus; 2A^PRO^, 2A proteinase; M, matrix; NS1, non-structural protein 1; Nsp3, non-structural protein 3; L, leader; Nup, nucleoporin.

Many viruses usurp or disrupt nuclear functions to benefit their replication and transmissibility. Moreover, viruses have revealed the identity of several cellular proteins important for nucleo-cytoplasmic trafficking, including the nuclear export factors CRM1 (chromosome region maintenance 1; also known as exportin 1, XPO1) and NXF1 (Nuclear export factor 1; also known as TAP, TIP-associated protein), which were both discovered during viral RNA export studies [3,4,5] (Figure 1). These two nuclear export receptors are manipulated by many DNA and RNA viruses to promote viral mRNA export and/or inhibit host mRNA trafficking. Multiple viruses, including some viruses that exclusively replicate in the cytoplasm, are known to restrict export of host mRNAs to the cytoplasm using different mechanisms (Figure 2); this restriction is key to inhibit host gene expression [6,7,8]. In immune competent hosts, these inhibitions are incomplete or inefficient since induction of antiviral responses often results in viral clearance. However, in immune deficient hosts or during viral infection with highly pathogenic strains, viral-mediated inhibition of host gene expression can be severely detrimental to the host. The mechanisms involved in host mRNA nuclear export inhibition by viruses will be discussed below with significant focus on influenza A virus (IAV) and vesicular stomatitis virus (VSV). This review will also discuss how immune response is involved and influenced by cellular mRNA nuclear export suppression by viruses.

## 2. Biogenesis and Nuclear Export of Cellular mRNA

mRNA must be properly expressed and processed prior to nuclear export for functional protein production. Transcription, mRNA processing and mRNA nuclear export are highly conserved processes. Notably, many significant studies have been conducted in both yeast and metazoan systems that are relevant to these particular biological processes, which have been extensively reviewed elsewhere [9,10,11,12]. Here, we will specifically discuss studies regarding mammalian mRNA nuclear export with a brief introduction to mRNA processing and how it is linked to export.

Following gene transcription from the DNA template, pre-mRNAs undergo capping, splicing and polyadenylation, yielding mature mRNA. Acquisition of a 5' 7-methyl guanosine cap protects pre-mRNA from degradation and facilitates nuclear export. Splicing and then polyadenylation result in capped, intronless, and polyadenylated mature mRNAs [13,14]. 

Loading of mRNA export factors on mRNAs requires capping and splicing. Factors involved in these processes recruit the transcription-export (TREX) complex that is essential for mRNA export [14,15,16,17]. The TREX complex is formed by the THO complex, which comprises proteins involved in transcription and export, in combination with two export factors [17,18,19,20]. The export factors associated with the THO complex include human UAP56 or HEL (Sub2 in *S. cerevisiae*), which is also involved in splicing [21,22], and REF (also known as Aly; Yra1 in *S. cerevisiae*), both of which facilitate mRNA export [17,23,24,25,26,27]. The ATP-bound form of UAP56 interacts with REF [28,29]. This stimulates the association of REF with mRNA, which enhances the ATPase activity of UAP56 [29]. REF then recruits the mRNA export receptor heterodimer NXF1-NXT1 (Ntf2-like export factor 1; also known as p15) to the mRNA [5,30,31]. NXF1 binds mRNA with higher affinity in the presence of REF, and a model has been proposed in which NXF1 binds directly to the mRNA handed over from various adaptor proteins [32]. Among the NXF1 adaptor proteins in addition to REF, are 9G8, SRp10 [32] and E1B-AP5 [33,34]. Mex67p and Mtr2p are the yeast homologs of NXF1 and NXT1, respectively, that function analogously, and therefore, this protein complex is highly conserved in eukaryotes and is necessary for cell survival [35,36]. The NXF1-NXT1 complex mediates export of bulk poly(A) mRNA in cells [32,37,38].

The NXF1-NXT1 heterodimer interacts with phenylalanine-glycine (FG) repeat domains on nuclear pore complex proteins (nucleoporins or Nups), such as Nup98 [34,39], which mediate the translocation of mRNPs (messenger ribonucleoproteins) through the nuclear pore complex (NPC). Nup98 has key roles in nuclear export of mRNAs and nuclear import and export of proteins [34,40,41,42,43,44,45,46,47]. Indeed, a robust block of Nup98, which interacts with key mRNA export factors, results in nuclear retention of bulk mRNA [34,40,41,43]. Rae1/mrnp41 (or Gle2p/Rae1p in yeast) is a nucleo-cytoplasmic shuttling mRNA export factor that facilitates mRNP docking onto Nup98, and a model has been proposed in which Rae1 functions to promote the recruitment of NXF1 to Nup98 at early stages of NPC translocation [40]. Indeed, Rae1 binds mRNPs and mediates their nuclear exit through the NPC [48,49,50,51,52] and interacts with both Nup98 [44,53] and NXF1 [40]. Furthermore, Rae1 directly binds Nup98 via its Gle2p binding motif, or GLEBS, which facilitates single-stranded RNA binding [44,48,54]. 

Once an mRNP reaches the cytoplasmic side of the NPC, it is released by the actions of the mRNA export factors Dbp5, Gle1 and IP6 [55,56]. Dbp5 and Gle1-bound IP6 interact with Nups [55,57,58,59,60,61,62,63]. ATPase activity of Dbp5 is stimulated by Gle1-bound to IP6, converting Dbp5 ATP-bound state to an ADP-bound state [64]. At this step, a subset of proteins are removed from the mRNPs, including NXF1 [65], which changes mRNP composition and confers transport directionality [64,66,67]. Notably, a role for mRNA export factors in translation has also been indicated for NXF1 [68] and Gle1 [69]. 

In addition to NXF1, CRM1 is a critical protein involved in nucleo-cytoplasmic trafficking. CRM1 is an exportin that is required to export proteins containing a leucine-rich nuclear export signal (NES). However, CRM1 is also a nuclear export receptor for mRNA, although it mediates trafficking of only subsets of mRNAs. CRM1 also transports other RNAs such as ribosomal RNAs (rRNA), signal recognition particle (SRP) RNA and uridine-rich small nuclear ribonucleoprotein particles (U snRNPs) to the cytoplasm. CRM1 does not directly bind RNA, and therefore, it requires adaptor proteins that bind RNA cargo destined for export. CRM1 recognizes NESs on RNA export adaptor proteins and also interacts with RanGTP to mediate mRNA export through the NPC [70,71]. 

A novel mechanism of nuclear export of large mRNPs was recently discovered involving nuclear envelope budding [72], which is similar to nuclear egress of herpesvirus virions [73,74,75]. This process appeared to be type A lamin dependent, yet independent of NPCs. Type A and B lamins comprise the nuclear lamina, a filamentous, structural component associated with the nuclear inner membrane, which regulates multiple nuclear processes [76]. Speese and colleagues used the *Drosophila melanogaster* model to examine the nuclear role of a C-terminal protein fragment of the Wnt-1 receptor, DFrizzled2 (DFz2), in neuromuscular junction (NMJ) differentiation. DFz2C (DFz2 C-terminal fragment) co-localized with lamin C, which was required for DFz2C nuclear lamina localization. Surprisingly, the authors noted that the DFz2C/lamin C foci contained RNA. Using microscopic analysis and oligo-dT fluorescent *in situ* hybridization (FISH), the authors observed the presence of poly(A) RNA within these large foci. RNA-containing DFz2C granules appeared to invaginate and traverse both the inner and outer nuclear membranes. Strikingly, nuclear budding of these DFz2C granules contained an RNA transcript encoding a protein that localizes to the NMJ, and therefore, nuclear envelope budding may be a mechanism used to specifically deliver mRNAs to distinct cellular sites for translation so that particular proteins are localized to areas where they carry out their functions. Hence, Speese *et al.* revealed a new mechanism of mRNP nuclear export that is not mediated by the NPC [72]. Whether viruses utilize this pathway for nuclear export is currently unknown.

## 3. Viral Mechanisms to Exploit Nuclear Export Receptors

Both CRM1 and NXF1 were identified as nuclear export receptors in studies focused on delineating retrovirus-mediated export of unspliced or incompletely spliced viral RNAs [3,4,5]. These three integral studies highlight the importance viruses have played in understanding general cell biology. The discovery of CRM1 as a nuclear export receptor was determined by identification of a nuclear export signal (NES) in the HIV-1 Rev protein, which binds CRM1 and is indispensable for export of unspliced or incompletely spliced HIV-1 RNA [3,4]. To facilitate viral RNA translocation to the cytoplasm through the NPC using CRM1, Rev protein encoded by HIV-1 and other lentiviruses recognizes a Rev-responsive element (RRE), a unique RNA secondary structure on many unspliced or partially spliced viral RNAs, such as those encoding gag, pol, env, vif, vpr and vpu [77] (Figure 1).

NXF1 was originally identified as an interacting partner of the herpesvirus saimiri TIP protein [78]. Retrovirus researchers aimed at defining the export mechanism of viral RNAs from type D retroviruses, which lack a Rev protein, discovered that NXF1 was responsible for facilitating release of type D retrovirus RNAs from the nucleus [5]. Originally, Pasquinelli *et al.* observed that the Mason-Pfizer monkey virus (MPMV) constitutive transport element (CTE), a *cis*-acting stem-loop RNA, supported nuclear export of unspliced or partially spliced viral RNAs utilizing a separate pathway than that of RRE-containing unspliced viral RNAs and Rev [79] (Figure 1). The authors noted that the CTE bound to two unidentified host proteins. Shortly thereafter, Gruter *et al.* performed a series of biochemical and cell biological experiments to show that type D retrovirus CTEs promoted export of unspliced or incompletely spliced viral RNAs by binding NXF1, a previously unidentified metazoan nuclear export receptor homologous to the yeast poly(A) mRNA nuclear export receptor Mex67p [5,35]. Whereas lentiviruses, such as HIV-1, utilize the CRM1 pathway to export viral mRNA, type D retroviruses and most DNA viruses exploit NXF1 to export viral mRNA [6,7,80]. 

Herpesviruses are DNA viruses well known to interact with different nuclear export factors. Herpesviruses co-opt many cellular proteins for replication within the nucleus and to transport viral transcripts to the cytoplasm for protein synthesis. An excellent example of a herpesvirus protein involved in nuclear regulation is ICP27, a well-studied protein encoded by the α-herpesvirus herpes simplex virus 1 (HSV-1). ICP27 interferes with several steps during mRNA processing and usurps cellular nuclear export pathways to promote nuclear exit of viral intronless RNAs. ICP27 is a nucleo-cytoplasmic shuttling protein [81,82,83,84] that inhibits cellular splicing [85,86,87] and facilitates viral mRNA export by hijacking the RNA export factor REF [88,89] and export receptor NXF1 [90,91,92]. REF directly interacts with NXF1 [5,30,31] and links splicing and nuclear export [16]. ICP27 recruits REF away from splicing sites to assist export of viral intronless transcripts [89], and both REF and NXF1 are involved in viral mRNA export facilitated by ICP27 [88,89]. Moreover, ICP27 directly interacts with NXF1, which is required for ICP27-driven viral mRNA nuclear export [90,91,92]. It has also been reported that HSV-1 exports viral mRNAs using both CRM1-dependent and independent pathways [93,94]. 

Viral proteins that share homology with ICP27 exist in other α-, β- and γ-herpesviruses. These homologs interact with members of the host mRNA processing and export pathways that could potentially interfere with cellular mRNA export. ORF57 (also known as Mta) from Kaposi’s sarcoma-associated herpesvirus (KSHV) interacts with REF to recruit the entire TREX complex to intronless viral RNAs leading to efficient export [95]. In addition, KSHV ORF57 utilizes a redundant export pathway when REF levels are depleted by siRNA, which involves another mRNA export adaptor UAP56-interacting factor (UIF) [96]. The authors also show that co-depletion of REF and UIF blocks ORF57-mediated export enhancement of viral intronless mRNAs showing the dependency of KSHV on host processing and export machineries to achieve optimal viral protein expression. ORF57 from herpesvirus saimiri also directs viral transcript export by binding REF to recruit NXF1 [97], as does Epstein-Barr virus (EBV) EB2 (also known as SM, Mta, BMLF1) [98]. IE4 from Varicella-zoster virus (VZV) exports mRNAs via the NXF1 pathway by interacting with SR proteins [99]. Cytomegalovirus (CMV) UL69 interacts with UAP56 to mediate viral mRNA nuclear export [100]. As expected, herpesviruses have evolved somewhat similar mechanisms to support nuclear export of their intronless transcripts. 

Although cellular mRNA nuclear export inhibition by herpesviruses has not been demonstrated, one can imagine that usage of REF, NXF1 and/or CRM1 pathways by herpesviruses may alter cellular mRNA export resulting in cellular poly(A) mRNA accumulation in the nucleus of infected or ICP27-expressing cells. However, ICP27 did not inhibit cellular mRNA export in microinjected *Xenopus* oocytes [88]. This lack of effect on mRNA export may be due to the fact that REF is dispensable for cellular mRNA export [101], that subsets of cellular mRNAs are blocked but not yet identified since systematic approaches were not used, or there was enough unsequestered NXF1 left to promote cellular mRNA nuclear export. Additional studies assessing bulk poly(A) mRNA export and export of subsets of mRNAs will address whether ICP27 targeting of REF and NXF1 affects host mRNA nuclear export. 

Another virus that perturbs host mRNA export receptors is influenza A virus (IAV), which is an RNA virus that replicates in the nucleus, a key feature of members from the *Orthomyxoviridae* family. Therefore, IAV encounters a similar problem as nuclear-replicating retroviruses and DNA viruses in which it must replicate and export its viral RNAs in the face of a mounting cellular immune response. The mechanism by which IAV mRNAs are transported to the cytoplasm remains controversial, similar to what has been observed for HSV-1 [88,89,93,94] and EBV [102,103] viral RNA export. Elton and colleagues demonstrated an interaction between CRM1 and IAV nucleoprotein (NP), which binds viral RNA to form viral ribonucleoproteins (vRNPs). In addition, they found that leptomycin B (LMB), a potent inhibitor of CRM1, induced nuclear retention of NP in virus-infected cells, indicating that CRM1 mediates nuclear export of vRNPs [104]. Furthermore, Watanabe *et al.* also reported inhibition of IAV vRNP nuclear export by LMB [105]. However, other studies argue that NXF1 has a role in vRNP nuclear export [106,107,108]. A report by Read *et al.* demonstrated that while nuclear export of certain IAV mRNAs depends on NXF1, other viral mRNAs are less dependent on the NXF1 pathway [109]. In these studies involving NXF1 and viral RNAs, the key experimental approaches employed siRNA knockdown of NXF1 and/or protein-RNA binding assays without chemical cross-linking of NXF1 to viral mRNAs upon infection. As previously demonstrated, knockdown of NXF1 leads to a major retention of poly(A) mRNA in the nucleus [32,37,38], which results in cytotoxic effects from inhibition of host gene expression without inducing immediate cell death. Since siRNA depletion of NXF1 has major pleiotropic effects on host gene expression, it is hard to interpret whether NXF1 is directly involved in export of viral mRNAs, or whether export of the viral mRNAs is influenced by a secondary effect of NXF1 depletion. 

To definitively demonstrate the binding of NXF1 to IAV mRNAs during infection, rigorous cross-linking experiments should be performed to affix NXF1 and RNAs followed by assessment for viral mRNA presence in NXF1-bound fractions. This experimental approach is necessary since RNA binding proteins can be promiscuous and reassort after cell lysis. However, it is still possible that IAV mRNAs use NXF1 in a different manner than the host to promote nuclear export of viral mRNAs. IAV may modulate aspects of mRNA export mediated by both CRM1 and NXF1 to facilitate nuclear release of particular viral mRNAs, meanwhile preventing export of cellular mRNAs.

Viral utilization of host nuclear export pathways may influence the efficiency of host mRNA nuclear export, whereby viral mRNA saturation of host mRNA nuclear export pathways may inadvertantly block cellular mRNA transit. Conversely, several viruses specifically target cellular export factors/receptors to interfere with cellular mRNA export. Given the need for viruses to overcome immune responses and compete with the host for access to the translational machinery, it is not surprising that many viruses employ mechanisms to dismantle host mRNA export (Figure 2). 

## 4. Inhibition of Nucleo-Cytoplasmic Trafficking by RNA Viruses

### 4.1. Influenza Virus Inhibits Host mRNA Processing and Nuclear Export

Influenza A virus (IAV) has implemented several unique strategies to interfere with host nuclear processes, including mRNA processing and export. One IAV strategy to subvert the nuclear machinery to promote viral mRNA synthesis is cap-snatching, a mechanism whereby IAV cleaves the 5' 7-methyl guanosine cap of cellular transcripts to prime its own viral mRNA synthesis [110]. The viral polymerase complex is not the only weapon IAV utilizes to disrupt cellular nuclear processes. IAV uses its multi-functional non-structural protein 1 (NS1) to target constituents of the mRNA processing and export pathways. IAV NS1 localizes to both the nucleus and cytoplasm to disrupt host functions [111]. Induction of type I IFN limits influenza virus replication [112]; thus, IAV NS1 represses IFN production by disrupting cytoplasmic signaling pathways (reviewed elsewhere [111,113]), as well as host nuclear processes.

A major pool of NS1 resides inside the nucleus. Early studies showed that NS1 blocked nuclear export of cellular mRNAs using different mechanisms to target multiple stages of mRNA maturation and export [114,115,116] (Figure 2). First, NS1 blocks cellular pre-mRNA splicing by binding to a stem-bulge region in U6 small nuclear RNA (snRNA) [117]. Second, NS1 binds and disrupts the 30-kDa subunit of the cleavage and polyadenylation specificity factor (CPSF30/CPSF4), inhibiting 3' end processing of cellular RNAs [118]. Third, NS1 binds the poly(A)-binding protein II (PABII; also known as PABPN1-poly(A)-binding protein nuclear 1), another component of the mRNA 3' end processing machinery. NS1 disrupts the localization and activity of PABII leading to accumulation of RNAs with short poly(A) tails that fail to be exported to the cytoplasm [119]. These mechanisms to impede cellular mRNA processing carried out by NS1 contribute to IAV-mediated inhibition of host gene expression. IAV mRNAs are unaffected by the NS1-mediated disruption of mRNA processing because viral transcript poly(A) tail synthesis is carried out by the viral polymerase complex [120,121]. Also, NS1 regulates splicing of the viral M1 mRNA segment, an effect that likely promotes IAV infection [122]. One possibility is that NS1 influences viral splicing by interacting with the cellular RNA binding proteins NS1-BP (NS1-Binding Protein) [123] and hnRNP K [124]. Therefore, viral mRNAs are efficiently processed prior to nuclear export, and viral mRNAs are not subject to NS1-mediated inhibition of mRNA nuclear export [104,105,108,109].

Given the strong inhibitory activity of host mRNA nuclear export by NS1 and the fact that a robust poly(A) signal is still detected inside the nucleus upon IAV infection or NS1 transfection, these observations indicated that various host mRNAs may have escaped mRNA processing inhibition. Further studies then revealed that IAV NS1 interacts with the mRNA nuclear export machinery to exert its inhibitory effects. In fact, NS1 was shown to physically interact with and form an inhibitory complex with mRNA export factors including NXF1-NXT1, Rae1 and E1B-AP5 in an RNA-independent manner [125] (Figure 2). This nuclear export inhibition was reverted by overexpressing these mRNA export factors in NS1-transfected cells. In addition, Nup98 levels were reduced in IAV infected cells, and low levels of Rae1 and Nup98 conferred greater cellular susceptibility to IAV-mediated cell death and an increase in viral replication [125]. Since Nup98 and Rae1 are induced by antiviral cytokines such as IFNs [53,126,127] and mRNAs encoding antiviral factors are retained in the nucleus of cells with reduced Rae1 and/or Nup98 [125], down-regulation of this pathway is likely a viral strategy to promote virus replication. One can speculate that NS1 may bind mRNA processing and export factors simultaneously (Figure 2) as these pathways are connected. In fact, NXF1 and Mex67p affect mRNA processing since depletion of either protein results in hyperadenylation of transcripts [128,129,130,131]. Additional studies are needed to clarify the spatial and temporal interactions between NS1 and mRNA processing and export factors. In summary, nuclear IAV NS1 protein blocks processing and nuclear export of mRNAs required to establish an antiviral state during infection by physically interacting with and disrupting the activity of key components of the 3’ end processing and mRNA export machineries.

### 4.2. Vesiculoviruses and mRNA Nuclear Export Inhibition

Vesiculoviruses, such as VSV, are negative strand RNA viruses from the family *Rhabdoviridae* that replicate within the cytoplasm. Upon infection with vesiculoviruses, host gene expression is quickly suppressed by the viral matrix (M) protein [132]. VSV M contains nuclear localization signals that mediate its nuclear import [133]. In the nucleus, VSV M protein arrests cellular RNA nuclear export by interacting with the RNA binding protein Rae1 [53] that forms a complex with the nucleoporin Nup98 [53,134]. This results in inhibition of mRNA and snRNA nuclear export [53,126,134,135,136,137] (Figure 2). The M protein-mediated RNA export inhibition was detected using multiple methods, such as oligo-dT *in situ* hybridization [53,126,134,135], nucleo-cytoplasmic fractionation followed by real time RT-PCR [137] and nuclear export assays performed in *Xenopus* oocytes [134,135,136,138]. A mutant M protein (alanine substitution for residues 52–54) that is deficient in Rae1 binding does not inhibit mRNA nuclear export [53,134]. Additionally, when the methionine residue 51 of VSV M is mutated to arginine (M51R), which was identified in a temperature-sensitive mutant strain of VSV (tsO82) [139], this mutant M does not inhibit nuclear transport and is not targeted to the NPC [136]. M protein interaction with the Rae1-Nup98 complex has also been reported to inhibit transcription [140] (Figure 2). However, host transcription still occurs since nuclear retention of bulk poly(A) RNA is detected upon M protein expression, indicating that polyadenylation has taken place after transcription, and that VSV employs post-transcriptional mechanisms to prevent host gene expression. The intranuclear complex of VSV M-Rae1-Nup98 likely regulates transcription of subsets of mRNAs, as would be predicted based on the role of intranuclear Nup98 in transcription, as discussed below [3,141]. Transcriptional studies involving cross-linking strategies and run-on assays are necessary to functionally identify the genes that are directly regulated by the M-Rae1-Nup98 complex. 

Rae1 is an essential protein as the Rae1 knockout mouse is embryonically lethal [142]. Babu and colleagues performed experiments with trophectoderm cells from E8.5 Rae1^+/+^ and Rae1^−/−^ outgrowths and showed that bulk mRNA export was not blocked in Rae1^−/−^ cells at this embryonic development stage, but cells did not survive beyond that stage in the absence of Rae1, demonstrating the requirement of Rae1 for fundamental cellular functions. However, nuclear export of specific subsets of mRNAs was blocked in Nup98 and/or Rae1 haplo-sufficient mouse embryonic fibroblasts expressing ~50% of Rae1 wild-type levels, as demonstrated by nucleo-cytoplasmic fractionation followed by quantitative RT-PCR [125]. Thus, Rae1 promotes efficient cellular mRNP nuclear export.

The VSV M-Rae1-Nup98 complex may interact with and block nuclear export of subsets of mRNAs that encode regulators of gene expression, an effect that would consequently induce bulk mRNA nuclear export block. Another possibility is that the Rae1-Nup98 complex regulates bulk NXF1-mediated mRNA export, and therefore, VSV M protein interactions with the complex could result in nuclear retention of the majority of poly(A) mRNAs. Thus, additional studies using genome-wide approaches will be important to identify the mRNAs that directly interact with the Rae1-Nup98 complex to further our understanding of VSV M protein functions. 

In addition to being localized at the NPC, a pool of Nup98 is found inside the nucleus [126,143], and it is involved in transcription of subsets of genes [144]. A new function for Nup98 as a post-transcriptional regulator has also been recently demonstrated, in which Nup98 stabilizes selected p53 target mRNAs [145,146]. This effect probably supports p53 tumor suppressor activity, further validating that Nup98 is important for regulation of cellular gene expression and homeostasis. Therefore, to disrupt host gene expression, viruses strategically target Nup98 and the Rae1-Nup98 complex. 

During mitosis, both Rae1 and Nup98 were shown to regulate spindle assembly [147,148], and VSV M protein inhibited mitosis progression via the Rae1-Nup98 complex [149]. This effect is meaningful for cancer cells, which have a high mitotic index, since VSV is an oncolytic agent that preferentially replicates and kills tumor cells rather than normal cells. This topic is reviewed elsewhere [150]. 

Thus, VSV-mediated inhibition of host mRNA export allows the virus to translate its few mRNAs in the cytoplasm while avoiding competition with host mRNAs for the translation machinery. Furthermore, the VSV M-mediated inhibitory effect reduces expression of mRNAs encoding antiviral factors [137], which likely promotes virus replication. VSV is extremely susceptible to IFN effects, and IFN can up-regulate the mRNA export factor Rae1 and Nup98/96 levels and revert the arrest of mRNA nuclear export induced by VSV M [53,126,127]. Nup levels are also modulated during the cell cycle, an effect important for T cell proliferation [149]. Interestingly, M protein inhibits CD1d trafficking in cells to prevent antigen presentation to natural killer T cells, which produce IFNγ [151]. Presumably, this inhibition would prevent up-regulation of cellular factors, such as Rae1 and Nup98/96, to culminate in reduced IFN signaling. While host cells promote expression of Rae1 and Nup98/96 through IFN signaling to overcome VSV infection, VSV compromises host gene expression using its M protein to target Rae1 and Nup98.

The inhibitory function of VSV M protein on mRNA nuclear export is conserved among M proteins from other vesiculoviruses, including chandipura virus (CV) and spring viremia carp virus (SVCV). These M proteins block export of both mRNAs and snRNAs, as well as slow down the nuclear transport of proteins. As observed for VSV M protein, mutation of a conserved methionine eliminated the inhibitory activity and prevented proper targeting of the M proteins to the NPC [138]. In sum, vesiculoviruses enlist their M proteins to down-regulate cellular mRNA trafficking to prevent proper host gene expression while promoting their own replication.

### 4.3. Picornaviruses Disrupt Nucleo-Cytoplasmic Trafficking

Viruses from the *Picornaviridae* family disrupt host cellular mechanisms in an effort to promote virus protein expression and replication. Picornaviruses replicate within the cell cytoplasm, and therefore, several picornaviruses disrupt nucleo-cytoplasmic trafficking of host proteins and mRNAs to likely reduce expression of antiviral factors and prevent competition with cellular mRNAs for the translation machinery. Poliovirus (PV), human rhinovirus (HRV), Theiler’s murine encephalomyelitis virus (TMEV) and encephalomyocarditis virus (EMCV) are among these picornaviruses known to interfere with nucleo-cytoplasmic trafficking by targeting Nups and/or nuclear transport factors [127,152,153,154,155,156,157,158].

Infection of eukaryotic cells with PV and HRV results in degradation of Nup62, Nup98 and Nup153 by the viral 2A proteinase (2A^pro^), leading to alterations in NPC architecture and disruption of both host protein nuclear transport and mRNA nuclear export [127,152,153,157,159,160,161] (Figure 2). During PV infection, localization of nuclear proteins is disrupted, and they are distributed throughout the cell [152,157]. However, this mislocalization could be restored when PV infected cells were treated with specific inhibitors of the viral 2A^pro^, suggesting that the mislocalization of nuclear proteins is associated with 2A^pro^ activity during infection [157]. Nup98 was shown to be partially degraded by PV 2A^pro^ [127,159], and Castello *et al.* also observed that a pool of Nup98 was mislocalized to the cytoplasm of 2A^pro^-expressing cells [127]. Moreover, ectopic expression of PV 2A^pro^ resulted in host mRNA nuclear export block likely due to the observed cleavage of Nups involved in mRNA export, such as Nup62, Nup98 and Nup153 [127]. However, results from Park *et al.* did not show mRNA export inhibition in PV-infected cells in which degradation of Nup98 was detected over time [159]. It is possible that the discrepancy between these two reports is related to experimental timing and the levels of Nup degradation. Due to Nup degradation, NPC permeability is also apparent during poliovirus infection, and nucleo-cytoplasmic trafficking of host proteins is affected [157]. In addition, HRV infection also results in degradation of nucleoporins such as Nup153, Nup214 and Nup358, by the viral 3C^pro^ (3C proteinase) resulting in increased NPC permeability leading to mislocalization of nuclear proteins [162]. These viral-mediated effects on Nups may impact cellular mRNA export as certain Nups are important docking sites for mRNPs, and Nups regulate mRNP translocation through the NPC [70].

Cardioviruses EMCV and TMEV 2A proteins lack proteolytic activity, contrary to the 2A proteins of enteroviruses PV and HRV [163]. Thus, EMCV and TMEV employ an alternative mechanism to exert nuclear mRNA export inhibition. Leader proteins (L) of cardioviruses EMCV and TMEV inhibit cellular mRNA nuclear export but lack known enzymatic activity [154,155,156] (Figure 2). EMCV L protein localizes at the NPC and binds Ran, altering the RanGDP/GTP gradient and resulting in abnormal nuclear transport of proteins and mRNA nuclear export block [156]. NXF1-mediated bulk mRNA nuclear export occurs independently of Ran; therefore, the nuclear retention of mRNAs induced by EMCV L may be due to inhibition of Ran-dependent nuclear import of proteins involved in mRNA nuclear export. However, CRM1-mediated nuclear export of subsets of mRNAs also requires Ran [70], which may account in part for the mRNA export block exerted by EMCV L protein targeting of Ran [156]. Of note, EMCV L protein compromises NPC integrity resulting in greater NPC permeability and misregulated protein efflux [158] possibly by phosphorylating Nups [164], similar to mengovirus [165] and TMEV [154,155]. Again, the effect of increased NPC permeability on mRNA export has not been specifically addressed, but it could be altered by virus-induced Nup modifications, similar to that described for Nup degradation above. TMEV L protein disruption of nucleo-cytoplasmic trafficking results in efflux of both nuclear proteins important for viral replication and IRF-3, whose nuclear localization is key for IFN signaling [166], thereby diminishing antiviral responses. This may be a common strategy imparted by cardioviruses to interfere with innate immune signaling. Overall, by altering the NPC structure and disrupting nucleo-cytoplasmic transport of host proteins and mRNA, picornaviruses suppress the host antiviral response leading to increased virus replication.

### 4.4. Rotavirus Inhibits Host mRNA Nuclear Export

Another RNA virus that targets nuclear export as a means to suspend host access to the translation machinery is rhesus rotavirus (RRV). RRV was recently shown to exert a nuclear export block of bulk poly(A) mRNA induced by the viral non-structural protein 3 (Nsp3) [167] (Figure 2). The authors demonstrated that bypassing nuclear export inhibition during infection allowed cells to overcome rotavirus-induced host shut-off. These results suggested that the rotavirus-induced nuclear export block of poly(A) mRNA was responsible for the inhibition of host translation during infection. Future studies of the *Reoviridae* family will be necessary to identify the mechanisms involved in this nuclear export inhibition, which may reveal new regulatory steps of the cellular mRNA export machinery.

## 5. Inhibition of Nucleo-Cytoplasmic Trafficking by DNA Viruses

### Adenovirus Inhibits Host mRNA Nuclear Export

Adenoviruses (AdV) are DNA viruses that replicate in the nucleus and induce mRNA nuclear export block during the late phase of infection to block host protein synthesis [168,169,170,171]. AdV early proteins E1B 55 kDa and E4 Orf6 work in concert to restrict host mRNA export during the late phase of infection while promoting viral mRNA export [168,169,170,171,172,173] (Figure 2). Furthermore, E1B 55 kDa inhibits nuclear export of poly(A) mRNA in yeast, which however, is not impacted by the absence or presence of E4 Orf6 [174].

Studies to date have not clearly defined how E1B 55 kDa, assisted by E4 Orf6, mechanistically disrupts host mRNA nuclear export. However, it was shown that E4 Orf6 and E1B 55K-mediated regulation of mRNA export requires E4 Orf6 ubiquitin ligase activity, demonstrating the importance of E4 Orf6-induced degradation of specific substrates as a key step for mRNA export regulation [173]. Subsets of host mRNAs are refractory to export inhibition by AdV, including Hsp70, β-tubulin, and IFN-induced mRNAs, and this effect is dependent on E1B 55 kDa [175]. Moreover, nuclear export of AdV mRNA requires the bulk poly(A) mRNA export receptor NXF1 [176], and hence, host mRNAs would likely be outcompeted for export by copious amounts of viral mRNAs. To further support the hypothesis that E1B 55 kDa may target NXF1 to block host mRNA export, AdV E1B 55 kDa interacts with the predominantly nuclear hnRNP host protein E1B-AP5 [33], which in turns binds to NXF1 [34]. Increased expression of E1B-AP5 facilitates both viral and host mRNA export to the cytoplasm [33], which further implies that E1B 55 kDa may disrupt NXF1-mediated mRNA nuclear export via E1B-AP5. E1B 55 kDa nucleo-cytoplasmic shuttling is dependent on CRM1 [177]. However, CRM1 does not mediate export of AdV mRNAs, and the impact of E1B 55 kDa on CRM1-mediated nuclear export of subsets of cellular RNAs is unknown [178,179,180]. Most investigations into AdV effects on nuclear export have focused around viral mRNAs and not host mRNAs; therefore, additional studies are required to clearly define how AdV promotes nuclear accumulation of host mRNAs. Overall, these data indicate that AdV hijacks the mRNA nuclear export machinery to selectively regulate temporal expression of viral mRNAs and inhibit host gene expression to promote viral replication.

## 6. Conclusions and Future Perspectives

Various groups have revealed mechanisms used by diverse viruses to inhibit cellular mRNA nuclear export to prevent both expression of mRNAs encoding antiviral factors and viral mRNA competition with host mRNAs for the translation machinery. Overall, this results in suppression of host protein translation. In addition, viruses have significantly impacted our understanding of cellular mRNA nuclear export and the critical players involved. 

Nuclear envelope budding of large mRNP complexes provides a new alternative for mRNA nuclear export. The regulation of this novel mRNA export route remains undefined. Experiments were carried out in *Drosophila* [72], so whether mammalian mRNPs utilize this mode of export is unknown. If this mechanism is conserved, it is highly likely that many viruses will target or utilize this pathway. Indeed, herpesviruses exit the nucleus using a nearly analogous pathway by traversing the inner nuclear membrane through budding and acquisition of an envelope, followed by de-envelopment during fusion with the outer nuclear membrane to release naked capsids into the cytoplasm [181]. Whether viruses inhibit nuclear envelope budding to down-regulate host mRNA nuclear export will be of great interest.

Lastly, our understanding of viral disruption of cellular mRNA nuclear export pathways has potential therapeutic value. The utility of IAV NS1 and its potent effects on cellular mRNA export were recently used to identify small molecules that could antagonize NS1 functions that could be useful as antivirals [182,183]. A high throughput screen was performed using a chemical library, and each compound was tested for its ability to revert the NS1-mediated inhibition of host gene expression. This screen successfully identified a pan-virus inhibitor that down-regulated the mTORC1 pathway [182], which is required and activated during virus infection [182,184]. This chemical biology study also revealed the mTORC1 inhibitor REDD1 as a novel host defense factor [182]. An additional compound identified in the same screen inhibited pyrimidine biosynthesis, which reverted the IAV NS1-mediated host mRNA nuclear export block. The compound also effectively relieved the analogous block mediated by VSV M protein in the absence of virus via increasing NXF1 levels, which promoted mRNA nuclear export [137]. This study revealed a new connection between pyrimidine metabolism and mRNA nuclear export through regulation of NXF1 levels [137]. In addition, pyrimidine biosynthesis inhibited virus replication since it is critical for viral transcription, a process that is more robust than host transcription [185,186,187,188,189]. Thus, reversion of virus-induced host mRNA nuclear export block as a premise for antiviral screening can be used successfully to identify novel compounds or proteins that have therapeutic value for viral infections. 

In conclusion, studies of viral-host interactions with the nuclear transport machinery have provided important concepts for cell biology and viral pathogenesis. Future studies regarding nuclear export during viral infection should further enlighten our understanding of cellular mRNA export regulation and potentially identify new factors involved. 
